# Online Recognition of Daily Activities by Color-Depth Sensing and Knowledge Models

**DOI:** 10.3390/s17071528

**Published:** 2017-06-29

**Authors:** Carlos Fernando Crispim-Junior, Alvaro Gómez Uría, Carola Strumia, Michal Koperski, Alexandra König, Farhood Negin, Serhan Cosar, Anh Tuan Nghiem, Duc Phu Chau, Guillaume Charpiat, Francois Bremond

**Affiliations:** 1 INRIA Sophia Antipolis, 2004 route des Lucioles, BP 93, 06902 Sophia Antipolis, France; alvarogomezuria@gmail.com (A.G.U.); strumia.carola@gmail.com (C.S.); michal.koperski@inria.fr (M.K.); farhood.negin@inria.fr (F.N.); scosar@lincoln.ac.uk (S.C.); nghiemtuan@gmail.com (A.T.N.); chauducphu@gmail.com (D.P.C.); guillaume.charpiat@inria.fr (G.C.); 2 CobTek-Cognition Behaviour Technology, Université Nice Sophia Antipolis, 06100 Nice, France; a.konig@maastrichtuniversity.nl; 3 MUMC-School for Mental Health and Neuroscience, Alzheimer Center Limburg, Maastricht University, 6200 Maastricht, The Netherlands

**Keywords:** activity recognition, activities of daily living, assisted living, color-depth sensing, complex events, people detection and tracking, knowledge representation, senior monitoring

## Abstract

Visual activity recognition plays a fundamental role in several research fields as a way to extract semantic meaning of images and videos. Prior work has mostly focused on classification tasks, where a label is given for a video clip. However, real life scenarios require a method to browse a continuous video flow, automatically identify relevant temporal segments and classify them accordingly to target activities. This paper proposes a knowledge-driven event recognition framework to address this problem. The novelty of the method lies in the combination of a constraint-based ontology language for event modeling with robust algorithms to detect, track and re-identify people using color-depth sensing (Kinect^®^ sensor). This combination enables to model and recognize longer and more complex events and to incorporate domain knowledge and 3D information into the same models. Moreover, the ontology-driven approach enables human understanding of system decisions and facilitates knowledge transfer across different scenes. The proposed framework is evaluated with real-world recordings of seniors carrying out unscripted, daily activities at hospital observation rooms and nursing homes. Results demonstrated that the proposed framework outperforms state-of-the-art methods in a variety of activities and datasets, and it is robust to variable and low-frame rate recordings. Further work will investigate how to extend the proposed framework with uncertainty management techniques to handle strong occlusion and ambiguous semantics, and how to exploit it to further support medicine on the timely diagnosis of cognitive disorders, such as Alzheimer’s disease.

## 1. Introduction

Research on technologies for assisted living has been growing in demand due to the aging of world population and the increasing number of elderly people living alone. The task of automatic recognition of daily living activities plays a fundamental role in this scenario, since it may provide doctors with a deeper glimpse of people’s daily routine. However, this task is a challenging problem, far from being solved due to the unconstrained nature of real-life scenes, and the large intra-class variance of human activities (e.g., each person may have their own way of preparing coffee). The recognition of human activities has been explored from different sensor perspectives over the years, e.g., from ambient- [1,2] to visual-sensing [3,4], up to their combination [5]. Ambient sensing tends to equip the scene with several low-level sensors (e.g., microphones, presence and door contact sensors) and to monitor people activities by their interaction with (or disturbances in) the sensor network [1,2]. Although ambient sensing by low-level sensors has its advantages, like preserving people privacy, it may undermine the recognition and detailed description of complex activities by modeling them as a function of relatively simpler sensor states (e.g., kettle turned on, moved cup). As an alternative for low-level sensors, visual sensing focuses on the direct observation of people during the realization of activities [3,4,6], which fosters more detailed representations of activities. However, noise due to scene illumination changes and the estimation of 3D information from 2D data may degenerate the quality of vision systems using 2D video cameras and consequently degrade their performance.

**Figure 1:**
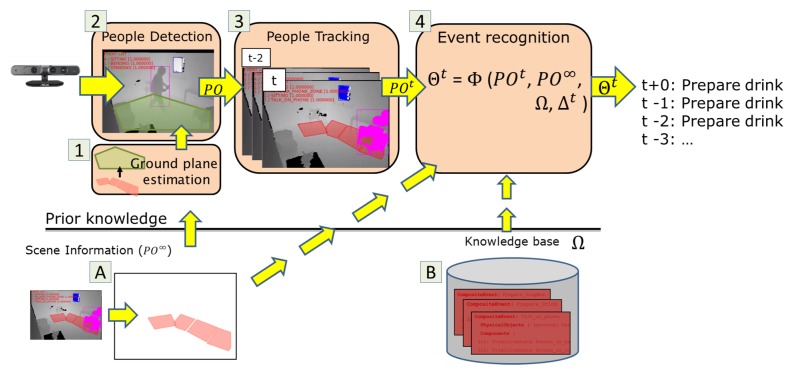
Knowledge-driven framework for visual event recognition. Firstly, (0) an estimation of the ground plane is computed using the vertexes of semantic zones; (1) Video frame acquisition is performed using a color-depth sensor. Then; (2) people detection module analyzes the video frame for instances of physical objects of type person. For each instance found, it adjusts its height using ground plane information; (3) Tracking step analyzes the set of detected people in the current and previous frames for appearance matching and trajectory estimation; (4) Event recognition takes as input the information from all previous steps and evaluates which event models in its knowledge base are satisfied. Recognized events are added to each tracked person’s history and the steps 2–4 are then repeated for the next frame. (A) Prior knowledge about the problem corresponds to semantic information about the scene geometry; (B) Knowledge base corresponds to the set of events of interest.

This paper proposes a fully working framework for event recognition based on color-depth sensing and ontological reasoning (Figure 1). It follows a person-centered pipeline (event recognition from people detection and tracking) to discriminate among the activities of different people and it explores the geometry of semantic zones to improve people detection and event recognition. The paper also extends the video event ontology language proposed by Vu et al. [7] from video surveillance to assisted living scenarios. Finally, it proposes an algorithm to improve people detection by coupling it with information about scene geometry (ground plane estimation using semantic zones). The rest of the paper is structured as follows: Section 2 presents related work, Section 3 describes the proposed approach, Section 4 describes the experiments carried out, Section 5 and Section 6 present the obtained results and discussion and Section 7 presents our conclusions.

## 2. Related Work

Knowledge-driven methods, like first-order logic and description-based models, provide a formalism to systematically describe domain knowledge about real-world phenomena using rules or constraints. Constraints provide a generic basis to combine different sources of knowledge [8,9]. They can be handcrafted by domain experts [3,4,8], learned from data or obtained by a combination of both forms [9]. Knowledge-driven methods are generally associated to an ontological formalism to define domain concepts and their interrelations [8,10]. Town [10] has introduced an ontological formalism for knowledge management and reasoning over raw visual data for video surveillance applications. Ceusters et al. [11] have proposed Ontological Realism to incorporate semantic knowledge into the recognition of high-level events using a video-analysis system supported by a human in the loop. Cao et al. [8] has used a rule-based engine to combine different sensing contexts (human and ambient) to monitor the daily activities of seniors. Human context (e.g., postures like sitting, standing, walking) comes from video camera data, while ambient context comes from inertial sensors attached to objects of daily usage and home appliances (e.g., TV remote control, and doors). In another direction, Chen et al. [12] have introduced a framework that combines ontology formalism for activity modeling with data-driven methods for model parameters update over time. Despite their representation power, knowledge-driven approaches are sensitive to noise due to their deterministic mechanism of reasoning. Therefore, these methods require that either their underlying modules for scene observation handle the sources of noise that intervene in the data [4,13] or that their reasoning mechanism is adapted to cope with noisy data at event level [14,15,16,17].

This paper focuses on the conception of a framework that associates a color-depth sensing pipeline for people detection and tracking with an ontology-driven mechanism of reasoning. Prior work on knowledge-driven methods and color-depth sensing (e.g., Asus Xtion PRO Live) have demonstrated the benefits of this sensing approach (3D information about the scene and invariance to illumination changes) to track the position of hands and facial features during psychomotor exercises (cognitive rehabilitation) [6], to recognize fall events in hospital rooms [13], and to recognize complex daily living activities of senior people (e.g., making the bed). Finally, Crispim-Junior et al. [3] compared the performance of event recognition between two different vision pipelines: a standard, color video camera and a color-depth sensor (Kinect^®^ with PrimeSense library). They have demonstrated that a pipeline with a standard color camera demanded a finer parameter tuning to handle low-level noise and achieve a performance comparable to the color-depth one. However, although the latter pipeline was less parameter-dependent, it could not detect people farther than 4 m, a limitation that may undermine its applicability in real-world scenarios.

The proposed event recognition framework differs from prior work in the vision pipeline adopted (people detection and tracking approaches) and the formalism for event recognition. Firstly, color-depth sensing tends to be more robust to noise due to scene illumination changes than conventional video cameras, and it allows the subsequent modules to solve 2D ambiguities by using 3D measurements of the scene. Moreover, the proposed vision pipeline employs an algorithm for people detection in color-depth sensing that extends the range of people detection from 3–4 m (e.g., Microsoft and Primesense libraries) to 7–9 m, by handling noise at depth pixel-level. Finally, the ontology-driven mechanism of reasoning allows to incorporate different sources of information efficiently, from common sense knowledge and event semantics up to dynamic information about visual entities. The combination of both modules enables one to model more complex and longer time-dependencies among events, barely explored before on online activity recognition.

## 3. Knowledge-Driven Event Recognition

The proposed framework is divided into the following modules (Figure 1): ground plane estimation (1), people detection (2), people tracking (3) and ontology-driven event recognition (4). ground plane estimation constructs a 3D estimation of the floor plane. People detection localizes people in every video frame. People tracking consists in finding appearance correspondences between people detected in the current and previous frames. Finally, event recognition combines the information of prior steps to infer which activities a person is performing. All steps follow an online fashion to address the task of continuous activity recognition in assisted living scenarios.

Next subsections describe the procedures employed to detect and track people in the monitored scene (ontology’s physical objects, Section 3.1, Section 3.2 and Section 3.3) and how to model and recognize complex activities of daily living using the ontology-driven approach (Section 3.4).

### 3.1. Ground Plane Estimation

The estimation of a ground plane is a key step for the vision pipeline, since its output is employed to improve the performance of the subsequent steps of people detection and tracking. The estimation process is made as follows: firstly, we search locally for pieces of planes, using the 3D-vertexes of the semantic zones. For each 3D vertex, we consider the cloud of points formed by its nearest neighbors and find the best plane which approximates it in the least square error sense (closed-form solution). When the approximation error is too high, i.e., when the local cloud of points is not flat enough, the plane is discarded. The obtained planes are clustered into larger planes weighted by the number of 3D points they possess. We compare any two pieces of planes during the clustering step based on the angle between their normals and on their alignment (distance between each center of mass and the other plane). We sort the newly obtained planes by their confidence (approximation error and number of points involved) and assign the first nearly horizontal plane to the ground plane. The ground plane is estimated using the 3D vertexes of semantic zones defined by the user around/in front of furniture objects.

### 3.2. People Detection

The people detection step is performed by the depth-based algorithm proposed by [18]. The algorithm performs as follows: first, background subtraction is employed on the depth image provided by the color-depth sensor to identify foreground regions containing both moving objects and potential noise. Foreground pixels are clustered into objects based on their depth value and their neighborhood information. Among these objects, people are detected using head and shoulder detectors. After this step, noise is removed using information from people detection and tracking from previous frames. At last, the background model of the background subtraction algorithm is updated using current step results. Given that the raw depth signal may be affected by the way certain materials reflect infrared beams, like some types of clothing materials [19], we have re-estimated people’s detection height by computing the Euclidean distance between the highest point in their silhouette (3D cloud of points) and the estimated ground plane (as a reference for the room floor level, Section 3.1). This procedure is required since lower limbs tend to be often missed due to either noise in depth measurement or to occlusion of limbs by scene furniture (e.g., desk). We have opted for a custom algorithm for people detection since the ones offered by the libraries of Microsoft and PrimeSense cannot detect people farther than 3–4 m away from the sensor. With our own algorithm we extend people detection to 7–9 m away, which is a more realistic distance for ambient assisted living scenarios. Finally, the people detection of background subtraction method does not make any assumption on people posture, and hence it can detect people in more unconstrained scenarios than the skeleton-based algorithm provided by Kinect^®^ standard SDKs [20].

### 3.3. People Tracking

The tracking algorithm takes as input the video stream and a list of detected people in a temporal sliding window. First, a link score is computed between any two detected people appearing in the time window using a weighted combination of six object descriptors: 2D and 3D positions, 2D object area, 2D object shape ratio, color histogram and dominant color. Hypothesis trajectories are built from links with scores greater than a pre-defined threshold. The reliability of each hypothesis trajectory is represented by the total score of its link scores. The trajectory of the objects are determined by maximizing objects’ trajectory reliability using the Hungarian algorithm [21]. Since the descriptor weights generally depend on the content of the video being processed, we use the control algorithm proposed by [22] to tune the weights on an online manner.

### 3.4. Ontology-Driven Event Recognition

**Figure 2:**
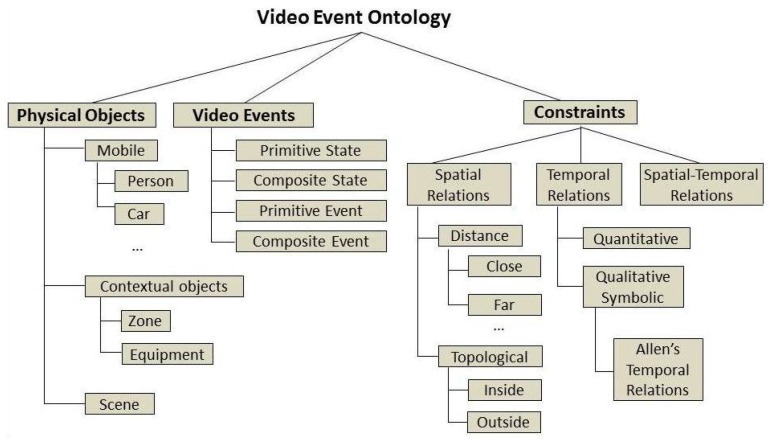
Video event ontology language. Three main concept branches are defined: physical objects, video events and constraints. Physical objects make abstractions for real-world objects. Video events describe the types of event templates available for activity modeling. Constraints describes the relations among physical objects and activities’ components (sub-events).

The proposed framework extends the declarative constraint-based ontology proposed by [7] with knowledge about activities of daily living, scene information and domain physical objects. The video event ontology language (Figure 2) employs three main conceptual branches: physical objects, events and constraints. The first branch, physical objects, consists in the formalization—at conceptual level—of the observations of the vision pipeline, i.e., the people and objects in the scene. The remaining two branches—video events and constraints—provide the basis for event modeling, i.e., the types of event models and the possible relations between physical objects and sub-events (namely components) that characterize a composite activity (or event).

Event models are defined by the triplet: physical objects, components (sub-events) and constraints; as described by Equation (1).

(1) $$\omega_{j}=\langle PO_{j},SE_{j},CO_{j}\rangle$$

where,

- $\omega_{j}$: event model *j*,
- $PO_{j}$: set of physical object abstractions $\left\{po_{j,1},..,po_{j,m}\right\}$ involved in model *j*, with $m=|PO_{j}|$,
- $SE_{j}$: set of components $\left\{se_{j,1},..,se_{j,k}\right\}$ in model *j*, with $k=|SE_{j}|$,
- $CO_{j}$: set of constraints $\left\{co_{j,1},..,co_{j,l}\right\}$ in model *j*, with $l=|CO_{j}|$.

**Figure 3:**
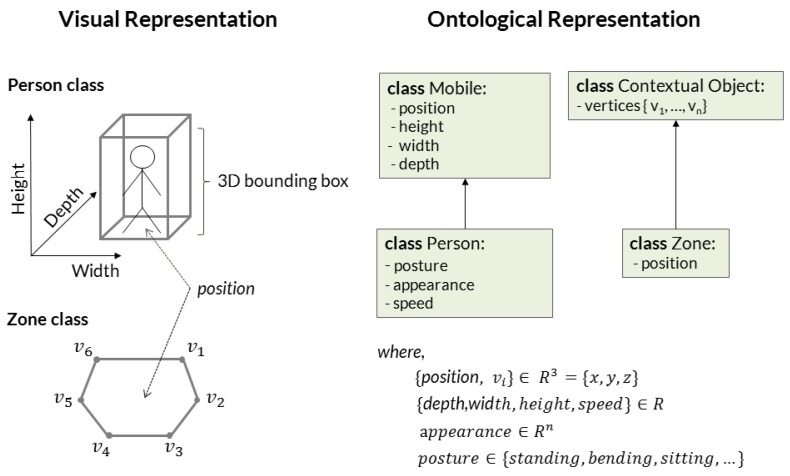
Physical objects integrate 3D visual information into the ontological events.

Physical object classes refer to abstractions of real-world objects that take part in the realization of target events (Figure 3). The possible types of physical objects depend on the domain for which the event modeling task is applied for. For assisted living settings, this paper defines five types of objects (Figure 1): mobile, person, contextual zone, contextual equipment and scene. Mobile is a generic class that contains the basic set of attributes for any moving object detected in the scene (e.g., 3D position, width, height, length). It is represented as a 3D bounding box. Person is an extension of Mobile class whose attributes are “body posture”, “speed” and “appearance signature”. Scene class describes attributes of the monitored scene, like the number of people in the scene. Instances of mobile and person classes are provided to event recognition step by the underlying vision modules (Figure 1, steps 2 and 3). Physical object’s instances are indexed by their time of detection *t* and an instance identifier *i*, i.e., $PO^{t}=\left\{po^{t,i},..,po^{t,n}\right\}$.

Contextual object class corresponds to a 3D polygon of *n*-vertexes that describe a piece of semantic information about the scene. Zones and equipment extend contextual object class and refer to knowledge about the scene (e.g., kitchen and couch zones or TV and table furniture, etc.). They may be obtained automatically by algorithms for scene discovery or be provided based on human knowledge. For instance, with the help of a software, one can easily define a 3D decomposition of the scene floor plane into a set of semantic regions, i.e., spatial zones (e.g., “TV”, “armchair”, “desk”, “coffee machine”). In the context of this work, semantic zones are provided as prior knowledge about the scene ($PO^{\infty }$) and their attributes are constant over time (non-temporal observations), since most semantic information about the target scenes refer to non-moving objects (e.g., furniture). Figure 2 demonstrates how the proposed framework integrates 3D information about the scene (prior and dynamic) as instances of physical objects.

Components are sub-events which describe pieces of semantic information relevant for the modeled event. They are used to describe knowledge that is shared by hierarchically higher event models. Constraints are used to define conditions about attributes of physical objects or between sub-events (components) of an event model. They are categorized into temporal and non-temporal constraints. Non-temporal constraints refer to conditions that do not directly depend on time, like spatial relations (e.g., in, close, out) and posture values (e.g., sitting, standing and bending). Temporal constraints refer to relations between the time intervals of an event model’s components. (e.g., BEFORE, MEET and EQUAL) [23] or about their duration.

Event models describe the relevant relations between the elements of the event triplet (physical objects, components and constraints). The ontology language provides model templates to support domain experts at modeling the hierarchical and temporal relations among events. Templates are categorized as follows (in ascending order of complexity):

- **Primitive State** models the value of a attribute of a physical object (e.g., person posture, or person inside a semantic zone) constant over a time interval.
- **Composite State** refers to a composition of two or more primitive states.
- **Primitive Event** models a change in the value of a physical object’s attribute (e.g., person changes from sitting to standing posture).
- **Composite Event** refers to a composition of two events of any type and it generally defines a temporal constraint about the time ordering between event components (sub-events).

Model 1 presents an example of composite event describing a temporal relation. The event model, “bed exit”, is composed of three physical objects (a person and two semantics zones) and two components. The components of the event, $c_{1}$ and $c_{2}$, are, model respectively, “the person position lying on the bed” and “the person being outside of the bed” (out_of_bed). The abstraction $p_{1}$ corresponds to a person’s instance dynamically detected by the underlying vision module. Contextual zones $z_{B}$ and $z_{SB}$ are abstraction for the semantic zones “bed” and “side of the bed”, which were *a priori* defined in the 3D coordinate system of the scene. The first constraint (MEET) defines that the time interval of component $s_{1}$ must start before the time interval of the component $s_{2}$ and both time intervals must briefly overlap. BEFORE relation, for instance, also expects the first interval to start before the second one, but different from MEET, it expects a time gap between them. The second constraint defines a lower bound to the duration of the sub-event out_of_bed, 3 s. Parameter values, such as minimum duration of an event model instance, are computed based on event annotations provided by domain experts.


**Model 1.** *Composite Event bed exit.*
*CompositeEvent(BED_EXIT,*
  *PhysicalObjects((p*_1_*:Person),(z_B_:Zone),(z_SB_:Zone))*
  *Components(*
    *(s*_1_*: PrimitiveState in_zone_bed (p*_1_*,z_B_))*
    *(s*_2_*: PrimitiveState out_of_bed (p*_1_*,z_SB_)))*
  *Constraints((s*_1_ *meet s*_2_*) // c*_1_
             *(duration(s*_2_*) > 1)) //c*_2_
  *Alarm ((Level : URGENT))*
*)*


Event inference (or recognition) is performed per frame *t* of a stored video sequence (or on the basis of a continuous video stream) using a sliding time window fashion. The inference step uses the temporal algorithm proposed by [7] for event recognition. For each time step *t*, the inference algorithm Φ takes as input the instances of physical objects present at *t* ($PO^{t}$), prior knowledge about the scene ($PO^{\infty })$, event history ($\Delta^{t-w}$) and the knowledge base to evaluate (Ω). The inference algorithm starts by evaluating the satisfaction of primitive states, since their constraints are only defined over instances of physical objects in the current frame (step#1). On step#2, it evaluates primitive and composite events that define constraints over instances of primitive states. On step#3, it evaluates the recognition of event models that define constraints over instances of events in lower levels of model hierarchy. Since the ontology language enables one to define a hierarchical structure among event models, inference step #3 is repeated until no model is satisfied by event instances from previous inference steps.

Given that event models are defined at conceptual level (using the event ontology language), the underlying vision pipeline can be fine tuned or replaced for a new scene without requiring changes to event models, as long as it keeps providing the same types of physical objects expected by the event models. Moreover, since the proposed framework stores event models as human-readable templates, it is very convenient to change models by adding new features or by adding models for new activities. For instance, when transferring the proposed system from location A to B, most changes refer to the update of contextual objects, i.e., the new location of semantic zones. Zone updates can be done in a couple of minutes using support software or be learned from labeled data. Supervised learning methods will require one to retrain all event classifiers once a new event class, input feature or dataset is considered. In the same situation, the proposed framework eases model addition and update, and by consequence, it facilitates knowledge transfer between different scenes (or datasets) with minimal changes.

## 4. Experiments

This paper has evaluated the proposed framework on three datasets: CHUN, GAADRD and nursing home. The first two datasets have compared the proposed framework to three variations of a state-of-art baseline for visual action recognition. The third dataset, nursing home, has evaluated the performance of the proposed framework on a more unconstrained scenario: the continuous monitoring of a senior person in her apartment, a scenario where only depth signal data is available. Software development was carried out using C++ language (version 11). Both experiments and development have used a PC workstation equipped with an Intel Xeon E2609@2.40 Gigahertz, with 16 GB of RAM memory and Fedora 19 as operating system. Next sub-sections describe with more details the activity modeling step, the baseline methods and the datasets used during the experiments.

### 4.1. Activity Modeling and Knowledge Transfer

For each target activity in CHUN and nursing home datasets, we have defined a model containing at least two sub-events, one that fires when the most frequent posture for the given activity is recognized, and one that fires when the person is inside/close to the most relevant semantic zone for the activity. A temporal constraint was added to capture the time ordering between the sub-events. A time duration constraint was also added to delay the recognition of the target activity until the instances of concerned sub-events are recognized for a minimum period of time. The knowledge above described was obtained by watching video examples of each activity, except for duration constraints that were learned from training data.

The ontology-driven approach followed by the proposed framework has made easy to port event models between the CHUN and GAADRD datasets, since they contain similar activities. For instance, baselines approaches had to be re-trained from scratch to be tested on GAADRD dataset. However, to test the proposed method on the new dataset, we had to mostly update the geometry of the semantic zones and the minimum duration of activities to match the characteristics of the dataset (e.g., “prepare drink” and “watering the plant” events only takes a few seconds in GAADRD contrary to CHUN dataset). The structure of event models and other semantics have remained unchanged. These changes were carried out in a matter of minutes after watching a video of a person performing the target activities in the GAADRD dataset. Since GAADRD dataset introduces a new activity, ”Turn on radio” we have followed the same procedure described for CHUN dataset to create a model for the new activity.

### 4.2. Performance Baselines

To compare the performance of the proposed approach with the state of the art, we have chosen the action recognition pipeline described in [24]. Support Vector Machines (SVM) for action classification trained with a bag-of-visual-word embedding over descriptors of dense trajectories features. In short, for each video sequence we have first extracted local spatio-temporal patches using dense trajectories’ detector. Then we have cut patches around each trajectory point as described in [24]. For each patch, we have computed standard descriptors: trajectory shape, HOG, HOF and MBH. Then we have used each of the latter three descriptors to create a bag-of-words (BoW) representation as an embedding function (with *k* = 4000). Finally, support vector machines with RBF kernel are used to classify the video representation into one of the target classes. Classifiers are learned on a supervised manner using video segments clipped from the original video sequence using ground-truth data. For online testing, the descriptors of a video are extracted over a temporal sliding window of size *W* (frames) with step size *T* and a minimal number of features extracted denoted as *M*. For each sliding window step we have extracted descriptors and apply BoW with SVM classifier given the number of detected features is equal or above *M*. Hyperparameters *W*, *T* and *M* were, empirically, set to 40, 15 and 20; respectively. A hold-out validation scheme is employed for training and testing the baseline classifiers. Baseline approaches were: Dense trajectories (DT) with Histogram of Gradients descriptor (HOG), DT with Histogram of Optical Flow (HOF) and DT with the y-component of Motion Boundary Histogram (MBH${}^{y}$). All results are reported on the tested set of the respective baselines.

### 4.3. CHUN Dataset

**Figure 4:**
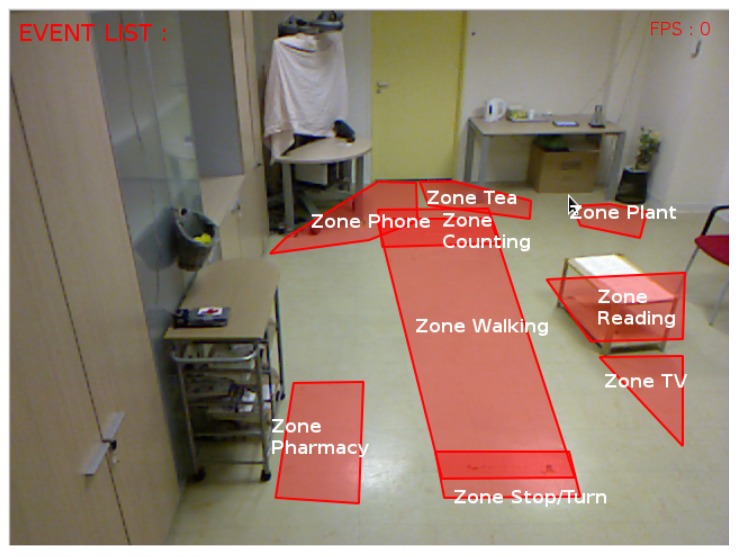
Contextual zones define geometric regions (red polygons, CHUN dataset) that carry semantic information about daily activities.

Participants aged 65 years and above were recruited by the Memory Centre of Nice Hospital to participate on a clinical study about Alzheimer’s disease. The study protocol asks the participants to carry out a set of physical tasks and Instrumental Activities of Daily Living (IADL) in a Hospital observation room equipped with home appliances (Figure 4) [25]. Experimental recordings used a color-depth camera (Kinect^®^, Microsoft^©^). The activities in the experimental protocol are divided into two scenarios: guided- and semi-guided activities. Guided activities (10 min) intend to assess kinematic parameters about the participant’s gait (e.g., walking 8 m). Semi-guided activities (∼15 min) aim to evaluate the level of autonomy of the participant by organizing and carrying out a list of IADLs. Semantic spatial zones are provided as prior knowledge about the geometry of the scene (Figure 4, red polygons): tea, telephone, plant, pharmacy, reading, TV, walking, stop/turn and counting. This evaluation focuses on the recognition of the following IADLs:

- Prepare drink (P. Drink, e.g., prepare tea/coffee);
- Prepare drug box (organize medication);
- Talk on the telephone (calling, answering);
- Read article;
- Search bus line and;
- Water the plant.

### 4.4. GAADRD Dataset

Participants aged 65 years and above were recruited by a Greek Institute under the scope of an European project for the study of Alzheimer’s disease, called Dem@care [26]. This dataset contains recordings of seniors carrying out physical tasks and IADLs in an observation room with similar settings to those adopted in CHUN dataset. Experimental recordings have adopted a color-depth sensor (Asus Xtion Pro Live^®^, 10 frames per second). We have focused our evaluation in GAADRD subset called DS8 which contains recordings of 25 seniors. In this subset, participants are asked to perform the following IADLs:

- Establish account balance (M.Payment);
- Prepare drink (P. Drink, e.g., prepare tea/coffee);
- Prepare drug box (P. Pill box);
- Read article;
- Talk on the telephone (T. Telephone, e.g., calling);
- Turn radio on; and
- Water plant.

We highlight that there are a few differences between IADLs of GAADRD and CHUN datasets. For instance, the “prepare pill box” activity in CHUN dataset consists in organizing a patient medication for a week, while in GAADRD dataset it corresponds to “taking the medicine”. Moreover, GAADRD introduces the activity “Turn radio on”. These differences have led to slightly different activity models between both datasets. Nevertheless, using the proposed ontological formalism we have swiftly adapted the event models’ definition of CHUN dataset to GAADRD. From here on in the paper we will refer to GAADRD-DS8 subset as GAADRD dataset.

### 4.5. Nursing Home Dataset

**Figure 5:**
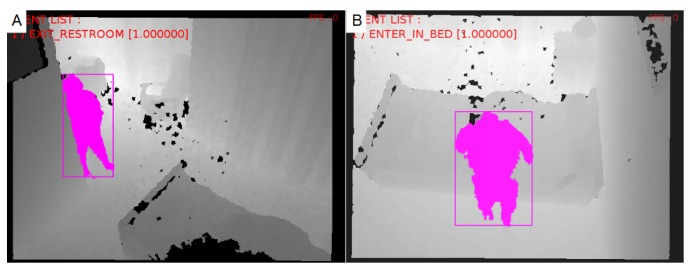
Monitored scene at the nursing home apartment: (**A**) living area camera displays an “exit restroom” event and (**B**) bed area camera displays an “enter in bed” event.

This dataset consists in 72 h of depth data recording about a 86 years old female, diagnosed with Alzheimer living in a nursing home apartment. Her apartment is monitored by two partially overlapping color-depth sensors. She displays agitation and aberrant motor behavior and the nursing home staff is interested in finding out more about her night time behavior, e.g., if she wanders during the night. In this evaluation we have focused on the performance of the proposed framework at describing common events in her daily routine: entering and exiting the bed, the restroom and the apartment; and sitting on the armchair. Figure 5 illustrates an example of the monitored scene. Since the nursing home dataset only contains depth signal recordings of the scene to anonymize the participant identity, the adopted baseline method, which is designed for color images, could not be computed for this dataset.

## 5. Results

This section summarizes the results of the evaluation carried out on CHUN (Section 5.1, GAADRD (Section 5.2) and the nursing home (Section 5.3) datasets.

### 5.1. CHUN Dataset

This experiment has evaluated the performance of the proposed framework on CHUN dataset (Table 1, Table 2 and Table 3). We have observed that the proposed approach has outperformed all variants of the baseline method and it has also presented the performance with the smallest standard deviation of the mean (Table 1). Among baseline variants, DT-HOG has the best performance (3/6 events) followed by DT-HOF (2/6). The performance values of the proposed framework have also generalized for the recognition of physical tasks (Table 2) and for IADL recognition on a larger cohort of participants (Table 3).

### 5.2. GAADRD Dataset

The second experiment has compared the performance of the proposed framework to baseline methods on GAADRD dataset (Table 4). The proposed framework has outperformed the baseline approaches in all event categories (Table 4). It has also presented the smallest standard deviation of the mean in performance. Results have also showed that HOF and MBH variants of the baseline method have completely failed to recognize the activities: prepare drug box, talk on the telephone and water the plant.

### 5.3. Nursing Home Dataset

Finally, the last experiment has evaluated the performance of the proposed method in the nursing home dataset. We have divided the results of this evaluation per point of views of the scene (bed or living room) and per day of recording (Table 5). In this experiment, event models make use of the physical object type “scene”. The scene object carries global information about the monitored scene and tracks its dynamics, like how the number of people in the scene varies over time. This type of concept is particularly useful to model the semantics of events related to entering/exiting the scene.

## 6. Discussion

This paper presented a fully working framework for visual activity recognition using color-depth sensing and knowledge-driven event models. This section summarizes the main findings of our evaluation ranging from the qualitative analysis of people tracking module up to the quantitative measurement of activity recognition performance on three datasets depicting seniors carrying out activities of daily living.

### 6.1. Overall People Tracking

To evaluate the quality of people tracking we have carried out an qualitative evaluation on the entire CHUN dataset and a subset of GAADRD dataset. The visualization of results has shown that in the short-term scenarios, such as the recognition of physical tasks, the tracking quality was nearly 100%. In mid- and long-term scenarios, like daily living activities and nursing home events, the tracking quality has dropped in cases of poor detection due to partial occlusion of a person’s body (e.g., person close to image borders or to scene furniture) or to the person be spending several minutes outside of the field of view of the sensor.

### 6.2. CHUN Dataset

The proposed approach has outperformed all variants of the baseline method and it has also presented the performance with the smallest standard deviation of the mean. Among baseline approaches, DT-HOG has the best performance (3/6 events) followed by DT-HOF (2/6). DT-HOG and DT-HOF variants of the baseline method have outperformed the proposed method on the activity ”Search bus line”. This happens because ”Search bus line” activity is carried out at the edge of the image and generally behind scene furniture. The resulting occlusion causes the step of people detection of the proposed framework to fail, which in consequence, causes event recognition to fail. However, since the adopted baseline method relies on a local representation of optical flow motion, and most of the person’s upper body motion is still visible, the performance of the baseline method is higher. The superior performance of the proposed method is mostly due to its capability to handle variable frame rate (here 4–15 frames per second) and to model the temporal dependencies between activity components. Since baseline methods rely on a temporal sliding window to capture temporal dependencies in test time, the information about short-duration activities is generally shadowed by the information of longer ones (e.g., the short “water the plant” versus the long “prepare drink”). The performance of the proposed framework (Table 2 and Table 3) can be also favorably compared to state-of-the-art approaches in a dataset with similar activities but different participants and camera setting [3]. Our framework has achieved a performance similar to prior work at the recognition of physical tasks (average recall: +1.12%, average precision: −4.6%). However, it had a higher precision for IADL recognition, which are more complex activities (av. precision: +4%). Finally, we have also observed that the performance of the proposed approach remains relatively stable as the size of the dataset increases (Table 1 × Table 3).

### 6.3. GAADRD Dataset

The proposed framework has also outperformed baseline approaches in this dataset and, as in CHUN dataset, it has presented the smallest standard deviation of the mean in recognition performance. Baseline methods have particularly failed to recognize the activities of “prepare drug box”, “water the plant” and “talk on the telephone”. These activities are particularly challenging because they have taken place either at the back of the scene or the edge of the image and their most discriminative feature is their localization. Since the amount of visible motion is very low, the decision of the classifier using HOF and MBH descriptors tends to be random. On the other hand, by using HOG descriptor, which describe edges, the corresponding classifier has achieved a higher performance than the other two descriptors for ”Talk on telephone” activity. Moreover, baseline methods were strongly affected by the low and variable frame rate of the dataset recordings (4 to 10 frames per seconds), a characteristic that the proposed framework can handle by focusing on relative temporal relations between sub-events and timestamp information instead of frames.

In summary, the ontology-driven formalism has a recognition performance that is superior to baselines with the great advantage of facilitating the transfer of event knowledge between different scenes, a important feature for real-world applications that baselines lack.

### 6.4. Nursing Home Dataset

In the nursing home dataset the proposed approach has also presented a high recognition performance (mean recall and precision are, respectively, 89.27% and 85.78% for living area events, and 91.66% and 86.35% for bed area events). We have observed that this performance generalizes across the monitored days, a fact which highlights the robustness of the proposed approach for more unconstrained scenarios. However, even though it has achieved a reasonable recognition performance in this unconstrained setting, a few challenges remain for future work. For instance, the low performance in “exit restroom”, “enter and leaving room” and “bed exit” events. This problem happens due to strong occlusion of the person’s body by walls, door frames (Figure 5a) and scene furniture (Figure 5b), like the bed. Missed instances of “exit bed” (see Model 1) refer to failures at people detection step that harm the recognition of the transition between a person “lying on the bed” to “standing” in front of it with legs occluded. To solve the reported cases, it is necessary to consider uncertainty estimates for the different steps of the vision pipeline and then reason accordingly to the scene geometry, a characteristic that the proposed method and state-of-the-art methods still lack.

### 6.5. Summary

The proposed framework outperforms baselines approaches on a variety of activities of daily living and on different datasets. Results also demonstrate that the performance of the proposed framework scales both for a larger number of participants and for unconstrained scenarios, like nursing home apartments. The demonstrated improvements come from addressing previously described limitations of event recognition using color-depth sensing [3], like the short-range field of view of the depth sensor; the underestimation of people’s body size—due to noisy depth signal and occlusion of body parts, and event reasoning on recordings with variable and low frame-rate. By handling these limitations, the proposed framework enables the modeling of longer temporal relations between events which are natural of real-life scenarios. The proposed framework can also distinguish among the activities of different people in the scene, a feature that is very important for assisted living scenarios and that state-of-the-art methods lack [24]. Currently, the reasoning mechanism in the proposed system is sensitive to noise in its input data, a problem which is mostly avoided by depth sensing and a fine-tuned pipeline for people detection and tracking. Future work will investigate how to extend the current reasoning mechanism with probabilistic features. Finally, the execution time of the event recognition framework is currently around 3.5 frames per second (people detection, tracking and event recognition), which enables a close to online monitoring of older people across most of the situations observed.

## 7. Conclusions

This paper has introduced and extensively evaluated a fully working, knowledge-driven framework for the recognition of daily activities of senior people in assisted living scenarios. The framework combines a constraint-based ontology language to model daily living activities with a robust pipeline for people detection and tracking on color-depth signals. The proposed framework outperforms baseline approaches and enables the modeling and recognition of longer and more complex events, natural to real-life scenarios. Moreover, the human-readable character of knowledge-driven models enables a person to easily understand the choices of the system, its mistakes and also facilitates the transference of the proposed framework between different scenes. The proposed system can be applied for any scenario for which color + depth and depth-only sensors are applicable and one can enumerate the features that characterize activities of interest. It excels at applications where one can make use of common sense knowledge and prior information to pinpoint relevant characteristic of events, and hence avoid acquiring several examples of target activities to generalize the same information from training data. The proposed framework is currently being used at a partner medical institute to support the daily evaluation of symptoms of Alzheimer’s disease; as a mean of interaction between a senior and serious games, and at the study of daily activities of seniors living at nursing home’s apartments and at their own residences.

Further work will investigate how to extend the proposed framework to handle uncertainty at low-level data and at semantic level, how to efficiently fuse multiple sensor data, and how to support the automatic diagnosis of cognitive disorders from behavioral data.

## Figures and Tables

**Table 1:** Recognition of IADLs—CHUN dataset—$F_{1}$-score.

Event	DT-HOG	DT-HOF	DT-MBH${}^{y}$	Proposed
Prepare drink	58.61	47.33	63.09	74.07
Prepare drug box	60.14	70.97	27.59	90.91
Read	51.75	56.26	65.87	83.33
Search bus line	66.67	63.95	42.52	60.00
Talk on telephone	92.47	46.62	72.61	95.00
Water plant	42.58	13.08	24.83	72.22
**Average ± SD**	62.0 ± 17.0	49.7 ± 20.3	49.4 ± 20.6	79.3 ± 13.0

SD: standard deviation of the mean.

**Table 2:** Recognition of a physical task in CHUN dataset.

IADL	Recall (%)	Precision (%)	$F_{1}$-Score (%)
Walking 8 m	90.75	93.10	91.91

N: 58 participants; 7 min. each; Total: 406 min.

**Table 3:** Recognition of IADLs in CHUN dataset.

IADL	Recall (%)	Precision (%)	$F_{1}$-Score (%)
Prepare drink	89.4	71.9	79.7
Prepare drug box	95.4	95.4	95.4
Talk on telephone	89.6	86.7	88.1
Water plant	74.1	69.0	71.5
**Average**	87.1	81.0	85.3

N: 45 participants; 15 min. each; Total: 675 min.

**Table 4:** Recognition of IADLs - GAADRD dataset - $F_{1}$-score.

Event	DT-HOG	DT-HOF	DT-MBH${}^{y}$	Proposed
Account Balance	44.96	34.71	42.98	66.67
Prepare Drink	81.66	44.87	52.00	100.00
Prepare Drug Box	14.19	0.00	0.00	57.14
Read Article	52.10	42.86	33.91	63.64
Talk on telephone	82.35	0.00	33.76	100.00
Turn on radio	85.71	42.52	58.16	94.74
Water Plant	0.00	0.00	0.00	52.63
**Average ± SD**	51.8 ± 34.4	23.6 ± 22.3	31.5 ± 23.3	76.4 ± 21.0

**Table 5:** Recognition of events in nursing home dataset.

Day	D1		D2		D3	
**Index**	Recall	Precision	Recall	Precision	Recall	Precision
**Camera at living area**						
Enter restroom	100.0	100.0	100.0	84.2	61.7	100.0
Exit restroom	100.0	34.8	100.0	41.0	100.0	81.4
Leave room	91.1	100.0	63.0	100.0	96.7	100.0
Enter room	79.7	100.0	61.1	100.0	98.3	100.0
Sit in armchair	100.0	100.0	87.5	100.0	100.0	45.4
**Average**	94.2	87.0	82.3	85.0	91.3	85.4
**Camera at bed area**						
Enter bed	100.0	100.0	100.0	62.5	100.0	77.8
Bed exit	50.0	100.0	100.0	100.0	100.0	77.8
**Average**	75.0	100.0	100.0	81.2	100.0	77.8

N: 1 participant, 72 h of recording per sensor.

## Abbreviations

The following abbreviations are used in this manuscript:

MDPI	Multidisciplinary Digital Publishing Institute
IADL	Instrumental Activities of Daily Living
